# Development of Embedded EM Sensors for Estimating Tensile Forces of PSC Girder Bridges

**DOI:** 10.3390/s17091989

**Published:** 2017-08-30

**Authors:** Junkyeong Kim, Ju-Won Kim, Chaggil Lee, Seunghee Park

**Affiliations:** 1 Department of Civil & Environmental System Engineering, Sungkyunkwan University 2066, Seobu-ro, Jangan-gu, Suwon-si, Gyonggi-do 16419, Korea; junk135@nate.com (J.K.); tolck81@gmail.com (C.L.); 2 School of Civil & Architectural Engineering, Sungkyunkwan University 2066, Seobu-ro, Jangan-gu, Suwon-si, Gyonggi-do 16419, Korea; malsi@nate.com

**Keywords:** tensile force estimation, embedded EM sensor, PS Tendon, B-H loop measurement, PSC girder

## Abstract

The tensile force of pre-stressed concrete (PSC) girders is the most important factor for managing the stability of PSC bridges. The tensile force is induced using pre-stressing (PS) tendons of a PSC girder. Because the PS tendons are located inside of the PSC girder, the tensile force cannot be measured after construction using conventional NDT (non-destructive testing) methods. To monitor the induced tensile force of a PSC girder, an embedded EM (elasto-magnetic) sensor was proposed in this study. The PS tendons are made of carbon steel, a ferromagnetic material. The magnetic properties of the ferromagnetic specimen are changed according to the induced magnetic field, temperature, and induced stress. Thus, the tensile force of PS tendons can be estimated by measuring their magnetic properties. The EM sensor can measure the magnetic properties of ferromagnetic materials in the form of a B (magnetic density)-H (magnetic force) loop. To measure the B-H loop of a PS tendon in a PSC girder, the EM sensor should be embedded into the PSC girder. The proposed embedded EM sensor can be embedded into a PSC girder as a sheath joint by designing screw threads to connect with the sheath. To confirm the proposed embedded EM sensors, the experimental study was performed using a down-scaled PSC girder model. Two specimens were constructed with embedded EM sensors, and three sensors were installed in each specimen. The embedded EM sensor could measure the B-H loop of PS tendons even if it was located inside concrete, and the area of the B-H loop was proportionally decreased according to the increase in tensile force. According to the results, the proposed method can be used to estimate the tensile force of unrevealed PS tendons.

## 1. Introduction

Recently, civil structures and their behaviors have become more complicated due to the development of materials, design, and construction technology. Also, the evaluation and maintenance of these structures have become very important. To inspect these structures, nondestructive tests (NDTs) have become a solution for evaluating structural health [1,2,3,4,5,6,7]. The main benefit of such non-destructive evaluation systems is that a structure does not need to be altered while being monitored. The condition of a structure can be assessed on site, while the information derived from a non-destructive evaluation can be instrumental in making engineering decisions concerning the fate of a structure. This ensures better judgment when determining whether or not a structure is safe, thus avoiding the construction, labor, and social costs of replacing a structure that actually does not require replacement. In addition, NDT techniques can be applied to new structures as part of a monitoring scheme, which leads to a better understanding of the behavior and performance during the construction and servicing of a structure.

Since the first post-tensioned concrete bridge was built in 1936, many PSC (pre-stressing concrete) bridges have been constructed globally [8]. However, after the sudden collapse of a number of post-tensioned concrete bridges, it was found that the post-tension system has long-term risks, such as corrosion of tendons caused by ingress of water and chloride ions into partially grouted ducts [9,10]. The tensile forces in the pre-stressing strands can vary due to a variety of losses including instantaneous losses such as elastic shortening, friction, and anchorage set occurring at the time of transfer of the pre-stressing force, as well as time-dependent losses due to steel relaxation and the concrete creep and shrinkage that occur after transfer of the pre-stressing force and during the life of the member. Accordingly, the measurement of the tensile force in a tendon becomes very important for long-term maintenance of such bridges, as well as for the purpose of design [11,12,13,14,15].

Various NDT methods have been studied to estimate tensile forces of tendons or cables. In addition, field measurements have been carried out by attaching sensors, such as a strain measuring gauges (Tensmeg), directly to the outside of the strand, or indirectly, by sensing the strain near the strand using an electrical strain gauge and vibrating wire strain gauge (VWSG) installed in the concrete or on a rebar near the duct [16]. More recently, various NDT methods for measuring pre-stressing forces have been studied using methods based on guided stress waves [17,18], a system identification technique based on modal parameters [19], an impedance method applied to an anchorage plate [20], and use of the in-strand encapsulated fiber Bragg grating (FBG) sensor [21,22].

Especially, a magnetic sensor is a reliable method for stress estimation of steel specimens, due to its outstanding superiorities including corrosion resistance, actual-stress measurement, nondestructive monitoring, and long service life [23]. The elasto-magnetic (EM) sensor is usually applied to measure the stress of ferromagnetic members and it consists of a primary excitation coil and a secondary induction coil. It can measure the permeability to estimate the stress and it has been used to monitor the stress in exposed steel cables on field more than ten years [24,25,26]. The many types of EM sensors were invested using coil type [27], yoke-shaped electromagnet with hall sensors [28], and coil type electromagnet with magneto-electric-laminated composite sensor [23].

However, the previous magnetic sensors should located closely to specimen and it cannot be applied actual PSC girder that the PS (pre-stressing) tendons did not expose to outside of girder. To overcome this limitation, this research proposed an embedded EM sensor that can embed into the PSC girder and measure the magnetic responses of internal PS tendons to improve field applicability. For this purpose, embedded EM sensors are developed for measuring the magnetic hysteresis loop variations in pre-stressing tendons and a test was carried out using a 6 m down-scaled PSC girder beam in order to verify the proposed method.

## 2. Development of the Embedded EM Sensor

**Figure 1:**
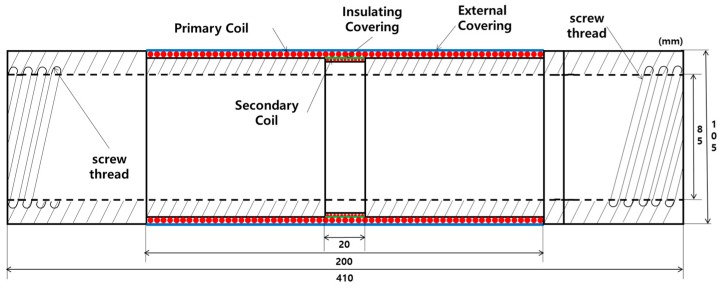
Schematic of the embedded EM sensor.

**Figure 2:**
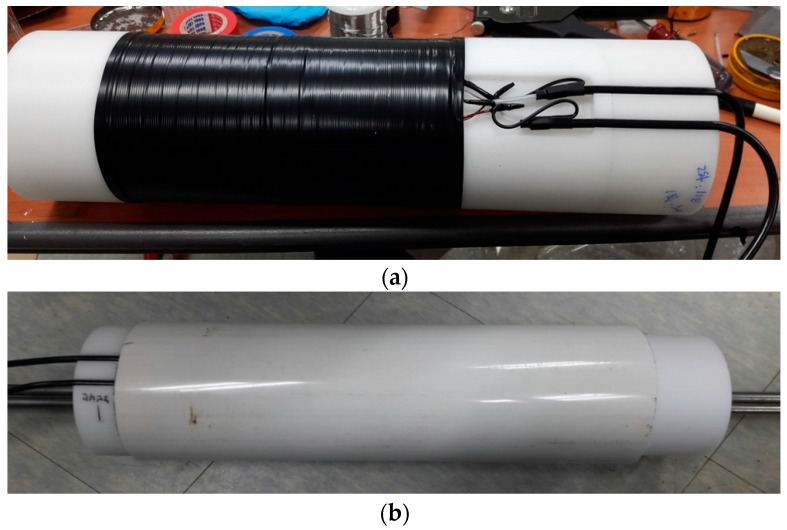
Embedded EM sensor. (**a**) Connection with coils and cable, (**b**) External protection cover.

This study applied an EM sensor to estimate the tensile force of PS tendons. The EM sensor can measure the induced stress of ferromagnetic specimen by the elasto-magnetic effect [29]. The EM sensor should be located inside a PSC girder to measure the magnetic responses of the PS tendons because the tendons are built into the PSC girder and could not approach after casting concrete. To apply an EM sensor to an actual PSC girder, an embedded EM sensor was developed. The embedded EM sensor consists of a cylindrical bobbin with screw threads on both ends to connect with the sheath, a primary coil to generate a magnetic field, a secondary coil to measure the magnetic response of the PS tendon, and an external cover to protect the coils, as shown in Figure 1. The ends of the primary and secondary coils are connected to coaxial cables (Figure 2a), and a PVC (poly vinyl chloride) pipe is used as the external cover (Figure 2b) to protect the EM sensor from impact and water from the concrete.

**Figure 3:**
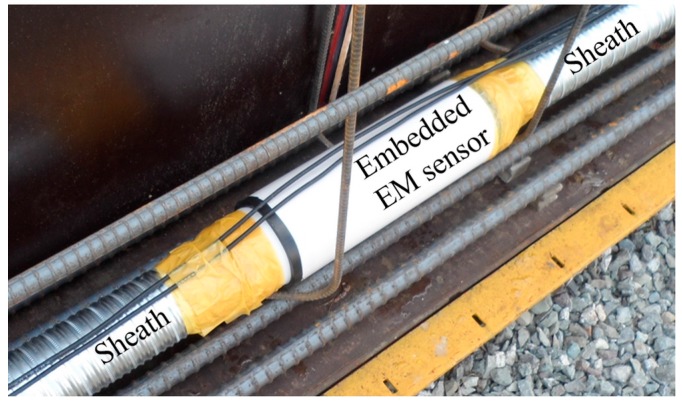
Installation of an embedded EM sensor.

The embedded EM sensor is installed between the sheaths as a sheath joint, as shown in Figure 3. Both sides of the EM sensor have screw threads, allowing the sensor to be installed at any position along the sheath line. Also, the joints are sealed using tape to prevent water permeation into the sheath.

## 3. Experimental Study

### 3.1. Experimental Setup and Test Procedure

**Figure 4:**
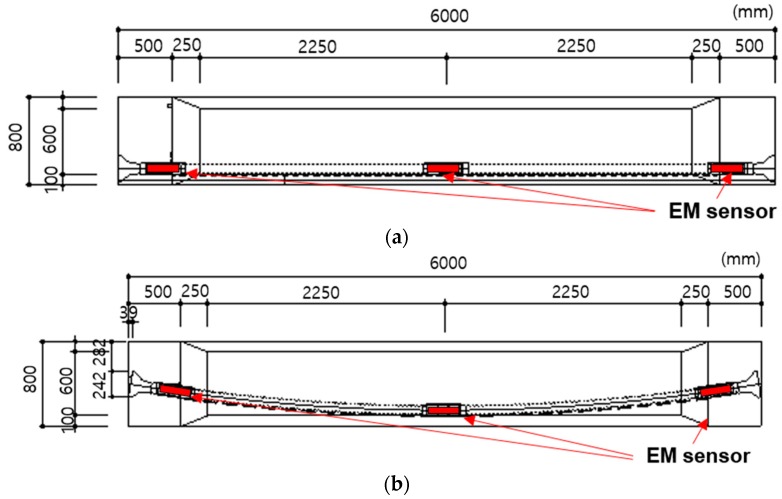
Schematic diagram of the test specimens. (**a**) Specimen 1: Straight sheath; (**b**) Specimen 2: Curved sheath.

**Figure 5:**
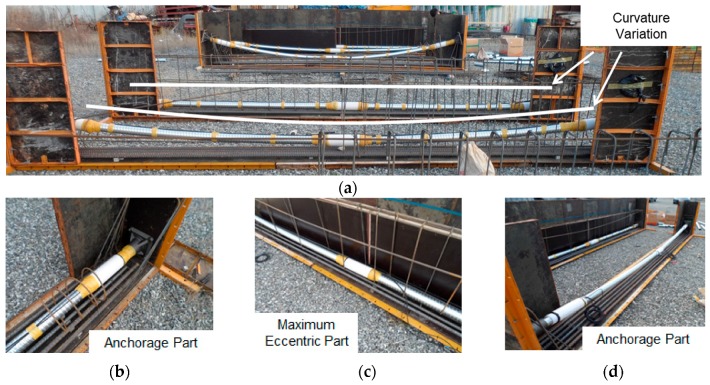
Installation of embedded EM sensors in a PSC girder. (**a**) EM sensor installed PSC girder, (**b**) Right anchorage part, (**c**) Maximum eccentric part, (**d**) Left anchorage part.

To confirm the proposed tensile force monitoring method, two down-scaled PSC girder specimens were constructed as shown in Figure 4. The span of the girders was 6 m, and the height was 1 m. The girders had 1 sheath line in each specimen (specimen 1 had a straight sheath, and specimen 2 had a curved sheath), and the embedded EM sensors were installed at the left and right anchorage parts and the maximum eccentric part as shown in Figure 4 and Figure 5.

**Figure 6:**
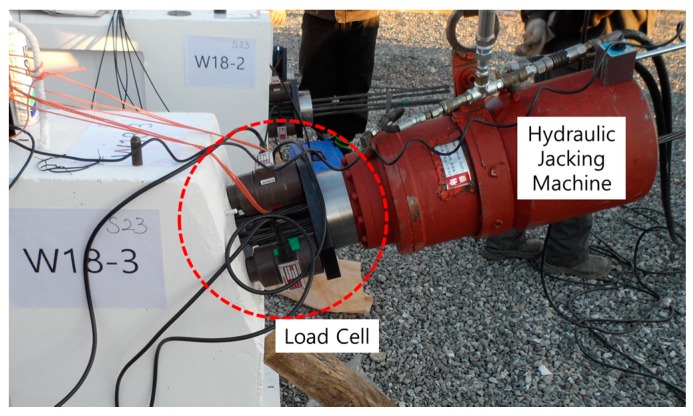
Installation of a load cell.

After installation of the EM sensors, the concrete was cast, and the PS tendons were arranged through the sheath lines. The nominal cross-section of each PS tendon was 158.8 mm, and four PS tendons were placed in each sheath line. To measure the actual tensile force, a three-point load cell was installed as shown in Figure 6.

The tensile force on the PS tendons was induced by a multi-tendon hydraulic jacking machine. The jacking step was divided into 6 steps, and the details are listed in Table 1.

### 3.2. Results of EM Measurement

**Figure 7:**
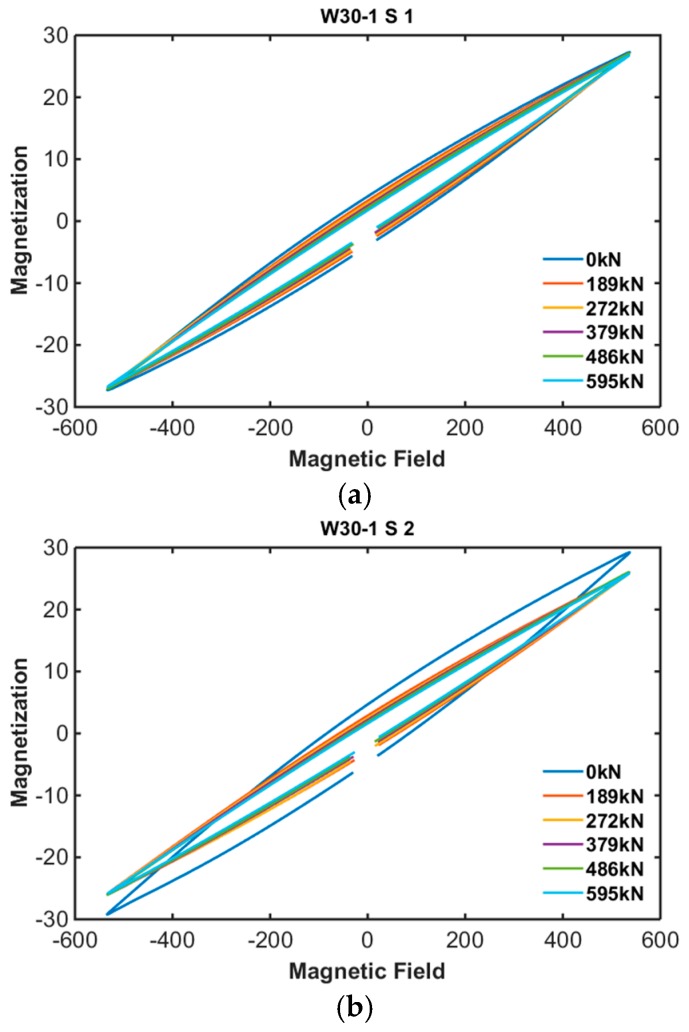

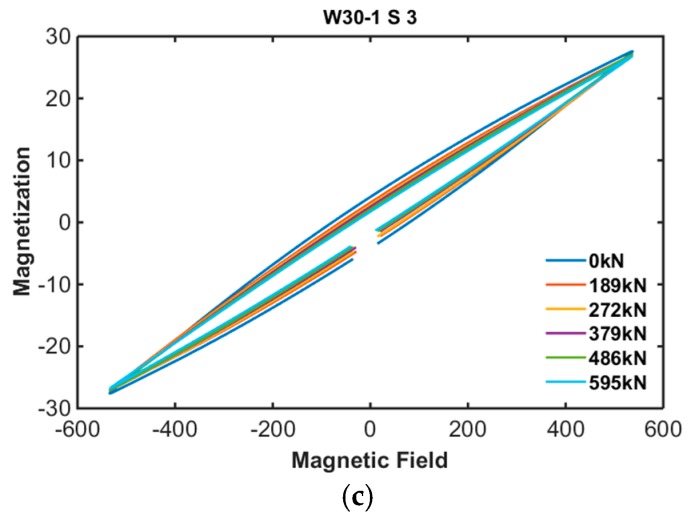
B-H loop of specimen 1: (**a**) Sensor no. 1-1; (**b**) Sensor no. 1-2; (**c**) Sensor no. 1-3.

**Figure 8:**
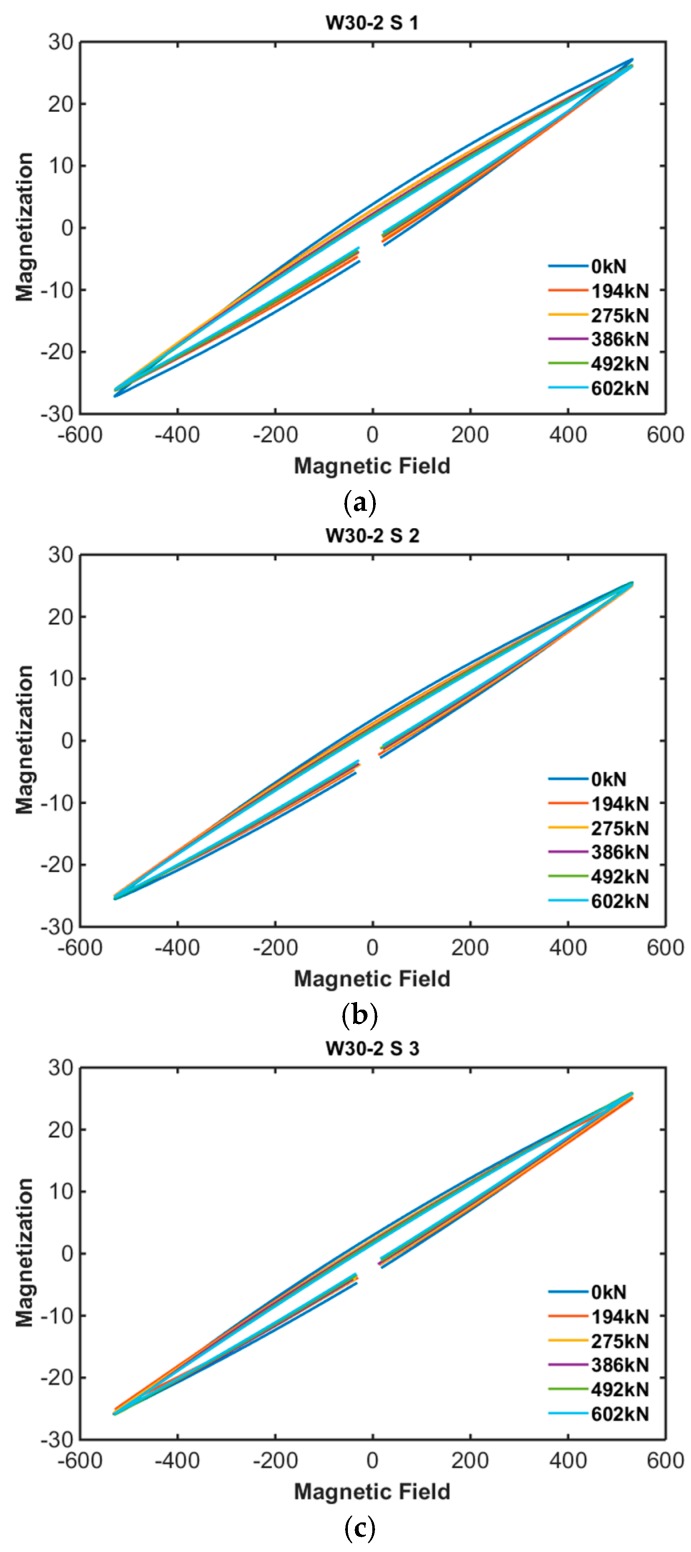
B-H loop of specimen 2: (**a**) Sensor no. 2-1; (**b**) Sensor no. 2-2; (**c**) Sensor no. 2-3.

The B-H loops of PS tendons were measured every jacking step using the embedded EM sensors. Figure 7 and Figure 8 show the results of B-H loop measurement of specimens 1 and 2.

According to the measurement results, proposed embedded EM sensor can measure the B-H loop of unrevealed PS tendon even if it located inside of concrete.

**Figure 9:**
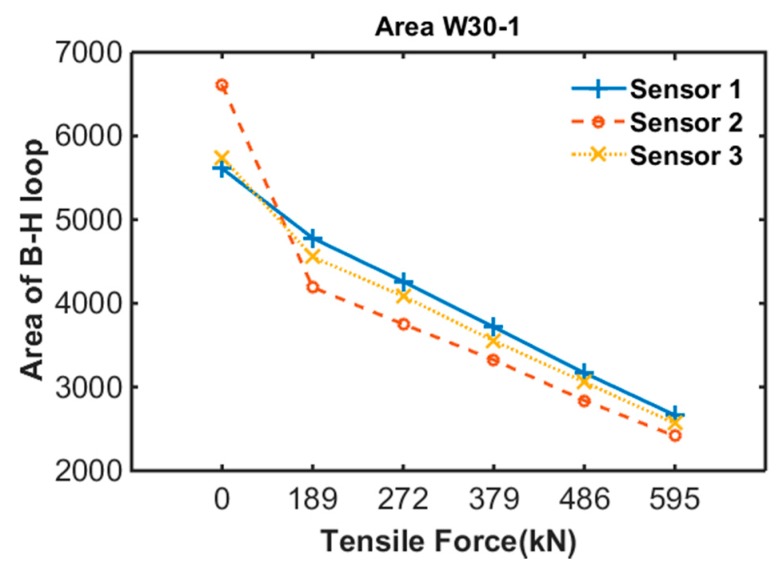
B-H loop of specimen 1.

To quantify the B-H loop variation due to induced tensile force, the area of the B-H loop was extracted. Figure 9 shows the variations in area of the B-H loop at each measurement of specimen 1. The results confirm that the area of the B-H loop decreased with increase in tensile force.

**Figure 10:**
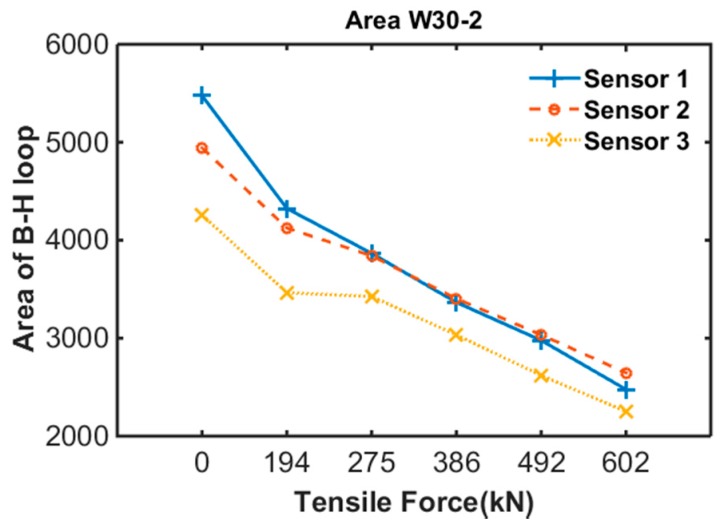
B-H loop of specimen 2.

Figure 10 shows the result of specimen 2. The results are similar to that of sheath no. 2; the area of the B-H loop decreased with increased tensile force.

To quantify the area variation due to tensile force, the area ratio was proposed as follows:

(1) $$A_{r}=\frac{A_{i}-A_{c}}{A_{i}}$$

where $A_{i}$ is the measured area of the B-H loop at a tensile force of 0 (step 1 of each specimen) and $A_{c}$ is the measured area of the B-H loop at the current step.

**Figure 11:**
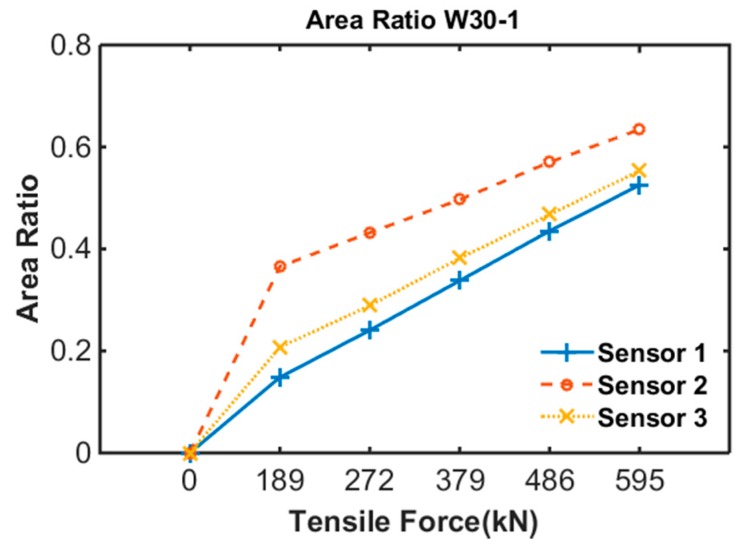
Area ratio of specimen 1.

Figure 11 shows the area ratio results of specimen 1. The area ratio increased with an increase in tensile force. The area ratio of Sensor no. 1-2 was larger than those of Sensor no. 1-1 and 1-3, indicating that the tensile force was concentrated at the center of the PS tendon. It caused by the sheath of specimen 1 was straight and there was no friction loss.

**Figure 12:**
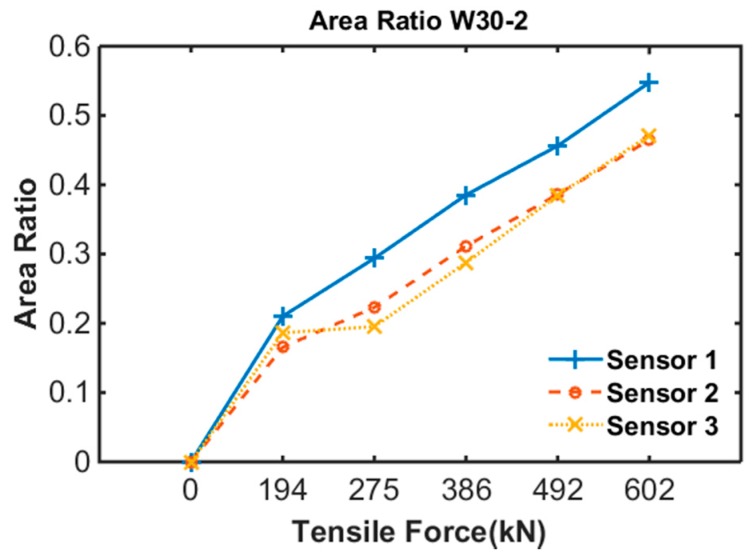
Area ratio of specimen 2.

Figure 12 shows the result of the area ratio calculation from the area of the B-H loop of specimen 2. The result shows a similar pattern to that of the result of specimen 1. However, friction loss was observed with the sensors because of the curved sheath of specimen 2.

**Figure 13:**
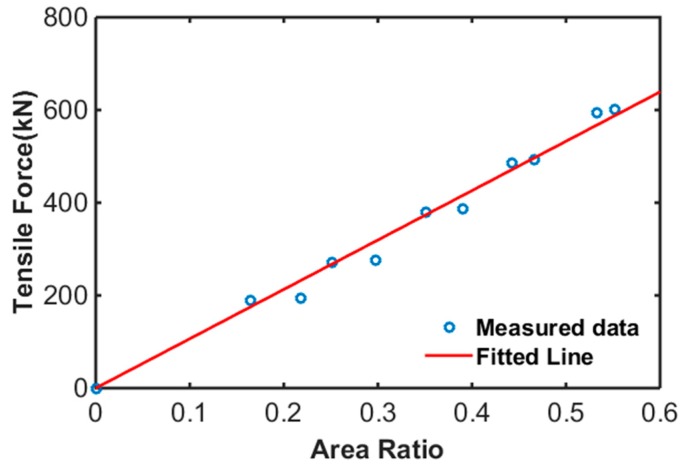
Relationship between area ratio and reference stress.

Figure 13 shows the relationship between area ratio of Sensor no. 1 of each specimen and reference tensile force measured by the load cell at the left side of each specimen. As shown in the figure, the area ratio and tensile force had a linear relationship, and the tensile force of a PSC girder can be estimated using Equation (2).

(2) $$Tensile\;Force\left(kN\right)=1065\;\times \;A_{r}$$

According to the results, the embedded EM sensors could estimate the tensile forces in PS tendons with acceptable error by tracking the area ratio variations. Furthermore, the friction losses and the tensile force distribution in the PS tendons could be monitored using the embedded EM sensor.

## 4. Conclusions

An embedded EM sensor based tensile force monitoring for PSC girders was proposed in this research. The B-H loop of ferromagnetic material is affected by the induced tensile force in the specimen. To measure the B-H loop of PS tendons in a PSC girder, an embedded EM sensor was developed as a sheath joint. To validate the proposed method, a down-scaled PSC girder test was performed using two PSC girder specimens that had 1 sheath line in each specimen. The embedded EM sensors were installed at the left and right anchorage parts and at the maximum eccentric part through the sheath before casting concrete. After casting and curing concrete, tensile force was induced in steps, and the B-H loop was measured at every tensile force step. Also, the reference tensile forces were measured from the load cell at the left anchorage of each specimen. The area of the B-H loop decreased with tensile force increase. To quantify the area variation, the area ratio was calculated, and the equation for estimating tensile force was derived by comparing the area ratio and reference tensile force. According to the results, the embedded EM sensor can measure the magnetic responses of unrevealed PS tendon even if it located inside of concrete and the tensile force could be estimated based on the area ratio variations of the PS tendon using the embedded EM sensors in the field environment.

## Figures and Tables

**Table 1:** Jacking steps of specimens.

Jacking Step	Tensile Force (kN)	
	Specimen 1 (Straight Sheath)	Specimen 2 (Curved Sheath)
1	0	0
2	189	194
3	272	275
4	379	386
5	486	492
6	595	602
